# Narrow-Band Ultraviolet B versus Oral Minocycline in Treatment of Unstable Vitiligo: A Prospective Comparative Trial

**DOI:** 10.1155/2014/240856

**Published:** 2014-08-21

**Authors:** Amir Hossein Siadat, Naser Zeinali, Fariba Iraji, Bahareh Abtahi-Naeini, Mohammad Ali Nilforoushzadeh, Kioumars Jamshidi, Parastoo Khosravani

**Affiliations:** ^1^Department of Dermatology, Skin Diseases and Leishmaniasis Research Center, Isfahan University of Medical Sciences, Isfahan, Iran; ^2^Department of Dermatology, Skin Diseases and Leishmaniasis Research Center, Students' Research Committee, Isfahan University of Medical Sciences, Isfahan, Iran; ^3^Skin and Stem Cell Research Center, Tehran University of Medical Sciences, Tehran, Iran

## Abstract

*Background*. We have compared NB-UVB and oral minocycline in stabilizing vitiligo for the first time.* Subjects and Methods*. 42 patients were divided equally into two groups: the NB-UVB and minocycline groups. Phototherapy was administered twice a week on nonconsecutive days. In the minocycline group, patients were advised to take minocycline 100 mg once daily. The treatment period was 3 months. Vitiligo disease activity (VIDA) score was noted every 4 weeks for 12 months. Digital photographs were taken at baseline and monthly intervals.* Results*. Before the therapy, disease activity was present in 100% of the patients, which was reduced to 23.8% and 66.1% by the end of therapy in the NB-UVB and minocycline groups retrospectively (*P* < 0.05). 16 of the 21 (76/1%) patients with unstable disease in the NB-UVB group achieved stability, whereas this was the case for only 7 of the 21 (33.3%) in the minocycline group (*P* < 0.001). The diameter changes were statistically significant at the end of treatment in the NB-UVB group compared to the minocycline group (*P* = 0.031). Side effects in both groups were mild.* Conclusion*. NB-UVB was statistically more advantageous than oral minocycline in unstable vitiligo in terms of efficacy and the resulting stability.

## 1. Introduction

Vitiligo is an acquired cutaneous disorder of pigmentation, manifested by the selective destruction of melanocytes in the skin, with a 1% to 2% incidence worldwide, without predilection for sex or race [1, 2]. There are some major hypotheses for the pathogenesis of vitiligo; the convergence theory is one. This theory states that stress, accumulation of toxic compounds, infection, autoimmunity, mutations, altered cellular environment, and impaired melanocyte migration and proliferation can all contribute in varying proportions to the etiopathogenesis of the disease [3]. High H_2_O_2_ level has been suggested to be responsible for the disappearance of melanocytes in vitiligo. Minocycline, an antibiotic possessing antioxidant activity, is capable of attenuating oxidative stress-induced neurotoxicity [2]. Song et al. showed that H_2_O_2_ decreases cell viability in a concentration-dependent manner which is attenuated by minocycline [2]. They suggest that minocycline may be used to prevent melanocyte loss in the early stage of vitiligo [2]. Since a causative treatment is not available, current treatment modalities are directed towards stopping the progression of vitiligo and achieving repigmentation in order to repair the morphology and functional deficiencies of the depigmented skin areas [4, 5]. The concept of stability in vitiligo is multifaceted, and no consensus has yet been reached on defining the criteria for this so far. An objective criterion, the vitiligo disease activity score (VIDA), was suggested by Njoo et al. in 1999 to follow the course of lesions. It is a 6-point scale on which the activity of the disease is evaluated by appearance of new vitiligo lesions or enlargement of preexisting lesions gauged during a period ranging from <6 weeks to 1 year [6] (Table 1).

Recently, minocycline has been proposed as an alternative therapy for unstable vitiligo [7]. But minocycline should be used with caution. Some of side effects of minocycline include light-headedness and vertigo, lack of concentration, gastrointestinal disturbance, increase of intracranial pressure, and unwanted skin and mucosal hyperpigmentation which should be considered before administration [2, 7]. To the best of our knowledge, this is the first instance of clinical evidence that compares the effectiveness of oral minocycline and NB-UVB in the treatment of unstable vitiligo.

## 2. Patients and Methods

A randomized clinical trial was done on patients with clinically diagnosed vitiligo vulgaris. The study included 42 consecutive patients of unstable vitiligo attending the clinic of Al-Zahra Hospital, a referral clinic of dermatology in Isfahan, Iran. The unstable vitiligo was defined as score 1–4 in vitiligo disease activity (VIDA) score [8].

Also the stability was defined as score 0, −1 in VIDA score. The patients were randomly, using a table of random numbers, allocated to one of two groups (NB-UVB or minocycline); 21 patients were thus allocated to each group. Reasons for exclusion were age ≤ 8 years or ≥50 years; pregnancy or intention to become pregnant; breastfeeding; other severe systemic diseases, for example, cardiovascular, renal, and hepatic failure; segmental vitiligo; acral vitiligo, taking any other vitiligo treatment within the previous 3 months; history of having taken any medication that could interact with minocycline (e.g., isotretinoin, oral contraceptive pills, etc.) within the previous 3 months; history of photomediated disorders such as systemic lupus erythematous and xeroderma pigmentosum (XP) and known hypersensitivity to the study medication. All patients provided written consent of informed participation beforehand.

### 2.1. Group A: The NB-UVB Group

All patients were treated with NB-UVB as monotherapy (V care UV therapy unit, Surya 440 ANB comprising Phillips Holland lamps with emission spectrum 311 nm, irradiance 1800–2000 *μ*W/cm^2^, calibrated twice yearly). Phototherapy was given twice a week on nonconsecutive days. Initial phototesting was not done. An initial dose of 0.25–0.75 J/cm^2^ was administered to all patients in the group. Standard photoprotection protocol for NB-UVB was observed. The optimal constant dose was achieved when minimal erythema appeared in the lesions. Otherwise, dose increment was carried out at the rate of 20% amount of the previous week. The phototherapy was continued until 100% repigmentation was achieved, or the treatment period was complete, whichever occurred earlier.

### 2.2. Group B: The Oral Minocycline Group

Patients were advised to take minocycline hydrochloride (MINOCIN) 100 mg once daily until 100% repigmentation was achieved or the 3-month treatment period was complete, whichever occurred earlier. During the study period, no other therapy was prescribed.

### 2.3. Procedures

In response to the treatment, using VIDA score, a 6-point score for activity evaluation of unstable vitiligo (Table 1) [8] was evaluated by observing the appearance of new lesions or any increase in the size of existing ones; repigmentation of existing lesions was also noted.

The treatment period was 3 months. During the study, the point of time at which stability was achieved was noted. Stability refers to no new and no increase in size of existing lesions for at least 3 months. On each visit, repigmentation was assessed and graded in the topographical area as follows. The patients were assessed every week 4 for 12 months by a blinded dermatologist. Baseline VIDA score was calculated, and disease activity was noted on each visit as follows.

### 2.4. Statistical Analysis

Statistical evaluation was done using SPSS for Windows version 16.0 (SPSS Inc., II, USA). Data were shown as frequency (percentage) or mean ± standard deviation (SD). The results of the study were analyzed by using “*t*-test,” *χ* ^2^-test of proportions.

## 3. Results

A total of 42 patients (24 females and 18 males) (range 15–44) in NB-UBV group (27.6 ± 9.4 years) and in minocycline group (25.4 ± 10.3 years, *P* > 0.05) were included in this study during a one-year period. All patients completed the study. The basic patient data and clinical characteristics of each group are summarized in Table 2.

All patients had stopped any method of therapy at least 3 months before entering the study, the NB-UVB group (21 patients), and the oral minocycline group (21 patients). Comparison of the demographic and disease parameters in the two groups showed no statistically significant difference in any of the variables (*P* > 0.05). 16 of the 21 (76/1%) patients with unstable disease in the NB-UVB group achieved stability (VIDA score 0, −1), whereas this was only 7 of the 21 (33.3%) patients in the minocycline group (*P* < 0.001) (Table 3).

In our study disease stabilization is more frequent in the NB-UVB group compared with the minocycline group (*P* = 0.019). There were patches of unstable vitiligo in all patients in both groups before the initiation of the study (*P* > 0.05). VIDA 3 or 4 (new lesions 3 months before) was seen in 11 of the 21 (52.3%) and 9 of the 21 (42.8%) patients in the NB-UVB and oral minocycline groups, respectively (*P* > 0.05). At the end of the therapy, activity was present in 5 (23.9%) and 13 patients (66.1%) in the NB-UVB and oral minocycline groups, respectively (*P* = 0.019). The difference in the percentage of patients showing activity at the start and end of therapy was statistically significant in both groups (*P* = 0.027). Of the five patients in the NB-UVB group with activity at the end of the study, three were initially unstable, whereas the remaining two were stable. The total mean size of the lesions in the NB-UVB group and the minocycline group changed from 25.68 cm^2^ to 14.20 cm^2^ and from 25.12 cm^2^ to 20.82 cm^2^, respectively. The difference in diameter changes was statistically significant at the end of treatment in the NB-UVB group compared to the minocycline group (*P* = 0.031). NB-UVB was generally well tolerated and adverse reactions were erythema and pruritus. Side effects were reported by 3 (14.2%) of the minocycline users. These included oral mucosal pigmentation, gastrointestinal complaint, and headache. All of these side effects were mild or moderately severe and no patient left the study due to side effects.

## 4. Discussion

In our study it was shown that, regarding NB-UVB versus oral minocycline in terms of stability, the former was more statistically advantageous with respect to the stability achieved in unstable vitiligo when assessed by the VIDA score. Most studies on the clinical aspects of stability in vitiligo have not been able to establish cut-off values that could be helpful in classifying the disease as active or stable in a patient. In this study, we use an objective measure: the VIDA score.

In the previous study by Parsad and Kanwar it has been suggested that oral minocycline was a new effective drug in the treatment of unstable vitiligo. They evaluated the efficacy of minocycline 100 mg once daily in 32 patients. They showed an arrest in the progression of disease in 29/32 patients and only three patients showed development of new lesions and/or enlargement of existing lesions. Ten patients showed arrest of depigmentation after 4 weeks of treatment. Seven patients showed moderate to marked repigmentation [7].

Recently Singh et al. performed a randomized controlled study to evaluate the effectiveness of dexamethasone oral minipulse (OMP) therapy versus oral minocycline in patients with active vitiligo vulgaris. They observed that, of the 25 patients in minocycline group, only 6 (24%) patients developed new lesions during 24 weeks of follow-up period, whereas in OMP group only 3 (12%) patients showed activity of disease [9]. These results in minocycline group were comparable to those observed in previous study by Parsad and Kanwar.

Our result shows that although the minocycline can have a role as a treatment for unstable vitiligo NB-UVB was more statistically advantageous.

NB-UVB therapy has emerged as one of the most effective treatment options in vitiligo over the last decade. A number of clinical studies have been conducted all over the world and all of these studies have documented a positive effect of narrow-band UVB therapy in vitiligo [6, 10, 11].

How narrow-band UVB therapy helps in vitiligo is not known with certainty but it has been postulated that NB-UVB acts in two different steps in vitiligo treatment. The first step is the stabilization of the depigmenting process and the second is the stimulation of residual follicular melanocytes [12, 13]. The stabilization of the depigmenting process is explained by the immunomodulatory effect of NB-UVB on the local and systemic immune responses [13, 14].

The limitations of the study include lack of blinding due to the nature of phototherapy, small sample size, and short treatment period. In summary, although the previous study of Parsad and Kanwar regarding treatment of unstable vitiligo by oral minocycline makes sense, the comparison of oral minocycline and NB-UVB in our study showed that the effect of oral minocycline is only attributable to the sunscreen effect and not quantitatively significant.

## 5. Conclusion

The results of our study reiterate the fact that minocycline is an effective and safe drug to control the activity of vitiligo but NB-UVB is more effective in reaching the activity of vitiligo comparable to oral minocycline. To the best of our knowledge, our study is the first to compare the efficacy and tolerability of oral minocycline with narrow-band ultraviolet B (NB-UVB) corticosteroids. In future studies, a comparison between minocycline and other modalities in the treatment of both stable and unstable vitiligo should be used to better compare treatment outcomes. In addition clarifying the exact mechanism of minocycline action in cellular biology is an issue that helps us design more accurate models of study.

## Figures and Tables

**Table 1:** Vitiligo disease activity score (VIDA): 6-point score for activity evaluation of unstable vitiligo [6].

Disease activity	VIDA score
Active in past 6 weeks	+4
Active in past 3 months	+3
Active in past 6 months	+2
Active in past 1 year	+1
Stable for at least 1 year	0
Stable for at least 1 year and spontaneous repigmentation	−1

**Table 2:** Demographics and disease parameters of the 42 patients.

	NB-UVB	Minocycline
Number of patients	21	21
Age in years (mean ± SD)	27.6 ± 9.4	25.4 ± 10.3
Sex (male/female)	8/13	10/11
Duration of disease before commencing therapy in years (mean ± SD)	15.13 ± 6.30	9.76 ± 3.84
Positive family history (*n*)	2	4
Skin type		
Type 3 (%)	16 (76.19%)	18 (85.71%)
Type 4 (%)	5 (23.81%)	3 (14.29%)
Mean body surface involved (mean ± SD)	30.5 ± 10.5	35.5 ± 11.5
Anatomical		
Head and neck	9	12
Trunk	13	7
Upper limb (proximal)	6	4
Upper limb (distal)	8	6
Lower limb (proximal)	5	6
Lower limb (distal)	4	3

**Table 3:** Stability achieved by using NB-UVB and minocycline in each group.

VIDA score	Beginning of therapy NB-UVB group	End of therapy NB-UVB group	Beginning of therapy Minocycline group	End of therapy Minocycline group
+4	6	0	4	2
+3	5	0	5	5
+2	4	3	6	4
+1	6	2	6	3
0	0	10	0	3
−1	0	6	0	4
